# Oculomotor behavior can be adjusted on the basis of artificial feedback signals indicating externally caused errors

**DOI:** 10.1371/journal.pone.0302872

**Published:** 2024-05-20

**Authors:** Frauke Heins, Markus Lappe

**Affiliations:** Institute for Psychology and Otto-Creutzfeldt Center for Cognitive and Behavioral Neuroscience, University of Münster, Münster, Germany; The Ohio State University, UNITED STATES

## Abstract

Whether a saccade is accurate and has reached the target cannot be evaluated during its execution, but relies on post-saccadic feedback. If the eye has missed the target object, a secondary corrective saccade has to be made to align the fovea with the target. If a systematic post-saccadic error occurs, adaptive changes to the oculomotor behavior are made, such as shortening or lengthening the saccade amplitude. Systematic post-saccadic errors are typically attributed internally to erroneous motor commands. The corresponding adaptive changes to the motor command reduce the error and the need for secondary corrective saccades, and, in doing so, restore accuracy and efficiency. However, adaptive changes to the oculomotor behavior also occur if a change in saccade amplitude is beneficial for task performance, or if it is rewarded. Oculomotor learning thus is more complex than reducing a post-saccadic position error. In the current study, we used a novel oculomotor learning paradigm and investigated whether human participants are able to adapt their oculomotor behavior to improve task performance even when they attribute the error externally. The task was to indicate the intended target object among several objects to a simulated human-machine interface by making eye movements. The participants were informed that the system itself could make errors. The decoding process depended on a distorted landing point of the saccade, resulting in decoding errors. Two different types of visual feedback were added to the post-saccadic scene and we compared how participants used the different feedback types to adjust their oculomotor behavior to avoid errors. We found that task performance improved over time, regardless of the type of feedback. Thus, error feedback from the simulated human-machine interface was used for post-saccadic error evaluation. This indicates that 1) artificial visual feedback signals and 2) externally caused errors might drive adaptive changes to oculomotor behavior.

## Introduction

Humans perform countless saccades each day. These rapid eye movements align the fovea with objects of interest and enable precise visual perception of our surroundings. Saccadic eye movements are crucial for collecting action-relevant information from the visual scene and are thus closely connected with intentions and upcoming actions [1]. Therefore, eye movements precede and accompany everyday behaviors like reaching or grasping [2,3], and they are indicative of our intentions or our locomotion direction [4–9]. To efficiently move the fovea to relevant locations within the visual scene and to obtain the information required to inform upcoming actions, saccades have to remain precise under changing conditions. Indeed, saccadic eye movements remain accurate despite of injury and disease [10–12], or fatigue [13], or across the lifespan [14,15]. Since saccades are too quick to be guided by visual feedback obtained during the ongoing movement, post-saccadic information is required to assess whether the saccade has brought gaze onto the intended visual target or whether a small secondary saccade is required to compensate for the discrepancy between the post-saccadic gaze position and the position of the target object. If the eye misses the target repeatedly and systematically, post-saccadic information is used to adapt future oculomotor behavior to the error, thereby restoring accuracy and efficiency [16–18]. However, adaptive adjustments to the oculomotor behavior even follow single isolated errors [19]. This learning process, termed saccadic adaptation, occurs under natural conditions [10–12], but can also be induced artificially by repeated and systematic intra-saccadic changes to the visual scene that evoke post-saccadic errors [20].

Post-saccadic error signals and expectations of the visual consequences of the saccade form the basis for postdictively estimating the proper motor command and adjusting the saccade such that post-saccadic errors are reduced [21]. Typically, it is assumed that the post-saccadic error is attributed internally, e.g. to an erroneous motor command that needs to be corrected. However, adaptive changes to the saccade amplitude still occur if the post-saccadic error is attributed externally. For example, human participants adapt their saccade amplitude in the double-step paradigm [20] regardless of whether they are aware of the error-inducing intra-saccadic target step or not [22–25]. In addition, participants are capable of gradually shortening their saccade amplitude using surface feature information about the target object to avoid a post-saccadic task error [26]. In that study, participants had to make an eye movement to a target object within an object array consisting of three geometric figures. The target object was defined by its color. Upon saccade onset, an intra-saccadic color change occurred, leading to a post-saccadic error as the eye did not land on the colored object. Participants gradually adjusted the saccade amplitude such that they avoided the post-saccadic error despite the intra-saccadic manipulation. The color change was clearly visible to the participant and thus would not have been attributed to an error in motor performance.

Implicit and long-lasting adaptive changes to the oculomotor behavior can also develop without a visual post-saccadic error signal. For example, saccade amplitude changes can be induced by reward [27], or if they improve the performance in a perceptual task [28,29]. These findings suggest that the error evaluation following a saccadic eye movement either requires a comparison between foveated and intended target object to assess whether the saccade was accurate [26,29–31] or information about whether the saccade was suited to meet the current behavioral goal [24,25,29]. Motor learning in general can be driven by both sensory errors and reward-based errors [32,33]. Sensory errors describe a mismatch between the expected and actual sensory consequences of a movement, resulting in participants adjusting their movement so that it produces the desired sensory consequences. Reward-based errors describe that participants do not achieve the task goal or do not receive reward despite performing a motor behavior that would typically be appropriate. Reward-based errors cause participants to adjust their motor command so that the movement is suitable to obtain reward or achieve a goal. Both are gradual processes that reach a similar adaptation magnitude and can lead to aftereffects [32,34,35]. Oculomotor learning in the absence of a visual post-saccadic error or in response to a post-saccadic task outcome shares a multitude of features with conventional saccade adaptation: 1) it develops gradually over time, 2) reaches a similar adaptation magnitude as conventional adaptation and 3) produces after-effects.

Previous work on oculomotor learning has mainly focused on the use of information sources naturally contained in the visual image, such as position or surface features of the objects in the scene. We wondered whether humans are also capable of using artificial feedback added to the post-saccadic scene to adjust their oculomotor behavior to task demands. The modulation of gaze behavior based on artificial feedback has a major application in human-computer interaction and assistive technology. Individuals with severe motor impairments, such as spinal cord injury, could use gaze to communicate action intentions to a human-machine interface that controls, for example, a robotic arm, a wheelchair or even an exoskeleton [36,37]. Such an assistive device could replace the motor function of paralyzed limbs and initiate intended actions such as reaching for a target object, thereby improving the users’ independence. To achieve this, it is crucial that the intention to act is decoded correctly from the gaze signal. Further, to ensure a safe use, the device must inform the user about the decoded action prior to movement onset so that the user can abort undesired actions following erroneous intention decoding. Providing this feedback visually after saccadic eye movements might be suitable to induce long-lasting adaptive modulation of gaze behavior due to post-saccadic error evaluation. The visually presented feedback could foster the users’ understanding of how the device decodes the action intentions from his or her eye movement behavior, potentially leading to an adjustment of the oculomotor behavior and improved intention decoding. More specifically, the feedback could also help the user to understand any systematic errors inherent to the system and to learn appropriate strategies to compensate for these externally caused errors. The feedback could either provide information about the inferred action intentions, e.g. about the object selected for an action, or it could provide information about the eye movement that serves as input for the decoding process of the device.

In the current study, we apply a new paradigm in which multiple objects are arranged in a circle and our participants actively select one object from all available objects. Thus, unlike in many previous eye movement studies, the target object is neither an isolated object on an otherwise blank computer screen, nor is it predetermined in any other way. Participants communicate the intended object via their gaze behavior to a simulated human machine interface. In different conditions, participants after the eye movement, receive visual feedback either about the decoded object or about the executed eye movement. In the action condition, feedback about the decoded object is provided by coloring the decoded object. Feedback about the decoded object informs the participants whether their eye movement was suitable to achieve the task goal. The extent of discrepancy between eye position and intended target is not available to the participant. In contrast, in the motor condition, feedback about the executed eye movement is provided by displaying the saccade end point. In this condition, the feedback about the executed eye movement does not convey explicit information about the task success, but contains precise information regarding the magnitude of the discrepancy between eye position and intended object. The participants were informed that bringing the motor feedback close to or on top of the intended target would lead to valid object decoding. In either condition, the goal of the task is that the simulated human machine interface correctly decodes the intended object and therefore a decoding error is avoided. To study whether participants use artificial feedback signals to adjust their oculomotor behavior to successfully achieve a task goal, we add a systematic distortion such that the feedback signal is not a veridical representation of the gaze behavior and thus of the intended object. Adding the systematic distortion introduces a decoding error and, accordingly, the need for adaptive adjustments in oculomotor behavior. The post-saccadic error is likely to be attributed externally and not to erroneous motor performance per se, as 1) the feedback is added to the scene only after the saccade and 2) the participants were informed that the human-machine interface might make errors and that they should learn to successfully interact with the system nonetheless. Thus, the current study aims to investigate whether adaptive changes to the oculomotor behavior occur even when the participants are explicitly told that the post-saccadic error might be of external nature, and whether feedback about the decoded action intention or feedback about the executed movement is better suited for eliciting the adaptive changes in oculomotor behavior that are necessary for decreasing the frequency of decoding errors.

## Method

### Sample

The sample consisted of 28 participants (16 female) aged between 18 and 49 years (*M* = 25.68, *SD* = 6.89). 25 participants were right handed (three left handed). Data were collected between May 24 and July 14, 2022. All participants had normal or corrected-to-normal vision and gave their informed consent in written form. The participants were compensated for their participation with either course credit or 8 €/h. Six participants were excluded from data analysis. Three of them misunderstood the task, in one case the calibration was not successful and one participant was excluded because the inclusion criteria for saccades were violated in more than 50% of the trials. For the sixth participant, 19 out of 20 saccades during the early learning phase did not meet the inclusion criteria and the participant was removed from further analysis in order to avoid basing the analysis on a single trial in the baseline.

### Experimental setup and conditions

The experiment was conducted in a dimly lit room in the Institute for Psychology of the University of Münster. The participants were seated at a 67-cm distance of an Eizo FlexScan 22-inch monitor (Eizo, Hakusan, Japan). The frame rate was set to 75 Hz and the screen resolution was 1152 × 870 pixels. Viewing was binocular while the right eye was recorded. The eye position was measured using the Eyelink 1000 eye tracker (SR Research, Ontario, Canada) at a sampling frequency of 1000 Hz. The experimental code was written in MATLAB 2018a (Mathworks, Natick, MA, USA) using the Psychophysics Toolbox extension [38,39]. A stable head position was ensured with a custom developed chin-forehead rest. The experimental procedures followed the 2008 declaration of Helsinki and were approved by the Ethics Committee of the Department of Psychology and Sport Science of the University of Muenster.

The aim of the study was to assess if participants can adjust their oculomotor behavior based on artificial feedback signals that are added to the post-saccadic scene, and to compare the effect of feedback about the eye movement itself (i.e. feedback about the primary saccade landing position) with the effect of feedback about the consequences of the eye movement (i.e. feedback about the decoded object). The condition during which participants were presented with feedback about their eye movement itself was termed “motor condition” and the condition during which participants received feedback about the decoded target object and thus the consequences of their eye movement “action condition”. All participants performed both conditions in separate recording sessions and the order of feedback type was counterbalanced across participants. At least 72 hours passed between the first and the second recording session of a participant. Each recording session lasted about 45 minutes, with about 15 minutes required for instructing the participant and further 30 minutes for the recording of eye movements. Each recording session began with 20 baseline circle-off trials, followed by 200 feedback trials and further 20 circle-off trials.

In both feedback conditions, participants were told that we were investigating how the intention to act is communicated via eye movements and how well this intention can be decoded by an artificial system. In the motor condition, the participants were told that we wanted to investigate how accurately our system computes the gaze position in order to subsequently decode action intentions, and that it was also investigated if participants can learn to deal with any error inherent to the system in such a way that the gaze position calculated by the system would actually be close to their desired interaction object. Their task was to ensure that the eye position computed by the system, indicated by a post-saccadic feedback signal, gets as close as possible to their intended object. In the action condition, the participants were told that we wanted to investigate how accurately our system decodes action intentions from gaze behavior, and that it was also investigated if participants can learn to deal with any error inherent to the system in such a way that the system would select the intended object. Their task was to ensure that the system selects their intended object as often as possible, indicated by the post-saccadic feedback signal.

### Stimuli

**Fig 1:**
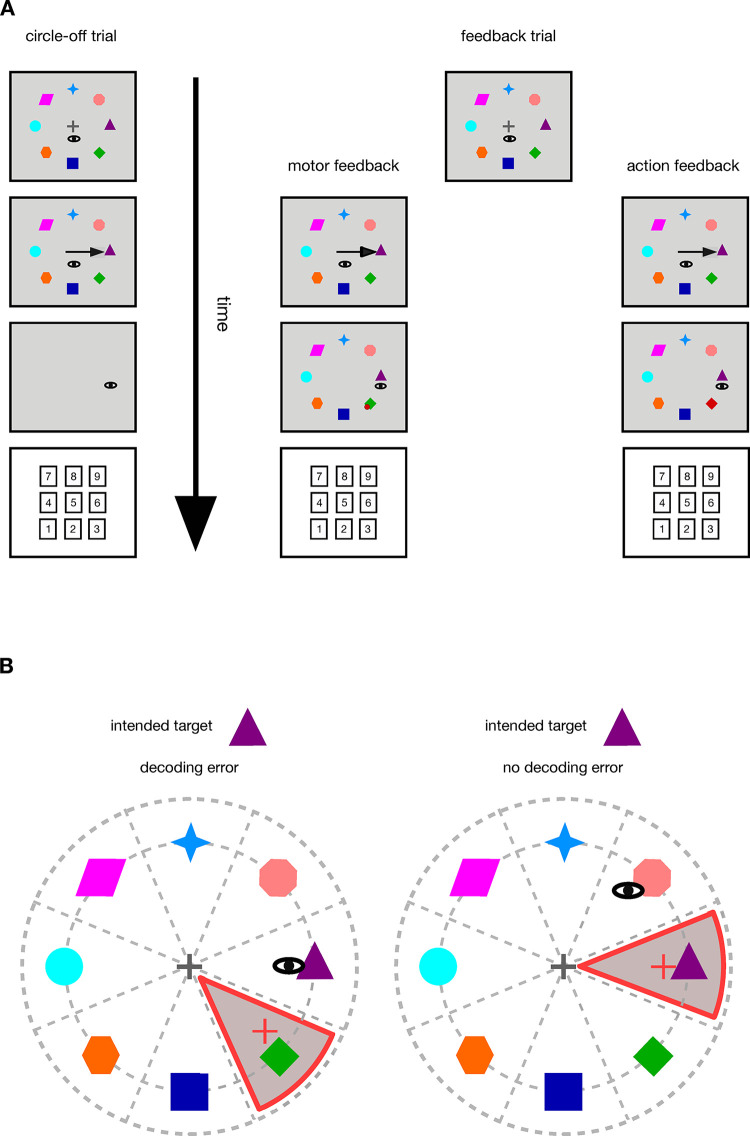
A) Sequence of events in the different trial types. In the circle-off trials, participants looked at the fixation cross. Upon removal of the fixation cross, they indicated the intended object in the respective trial via an eye movement to the system. Once saccade onset was detected, the objects on the circular path disappeared and the participants indicated the intended object with a key press. During feedback trials, participants looked at the fixation cross until it disappeared and communicated their intended target object via an eye movement to the system. Once saccade offset was detected, the primary saccade end point was calculated. In both feedback conditions, a clockwise 45 arc deg rotation was added to the actual end point of the primary saccade to simulate a systematic error inherent to the system. This distorted saccade end point was used for feedback presentation and object selection. In the motor condition, participants received feedback about the end point of their saccade, including the clockwise distortion. A red dot represented the saccade end point calculated by the system. In the action condition, participants received feedback about the object decoded by the system, also including the clockwise distortion. The object decoded by the system was colored red. After feedback presentation, participants indicated the intended target object with a key press. B) Trials with and without decoding error. In both cases the intended object was the purple triangle. On the left, the participant aimed the saccade at the purple triangle to indicate the intended target object to the system. Due to the 45 arc deg rotation added to the saccade end point, the distorted saccade end point fell in the target area of the green rhombus. The green rhombus was decoded and a decoding error occurred. On the right, the participant had adjusted to the 45 arc deg rotation and aimed the saccade toward the salmon octagon. Due to the distortion added to the saccade end point, the distorted saccade end point fell in the target area of the purple triangle. The intended object was decoded and no decoding error occurred.

**Fig 2:**
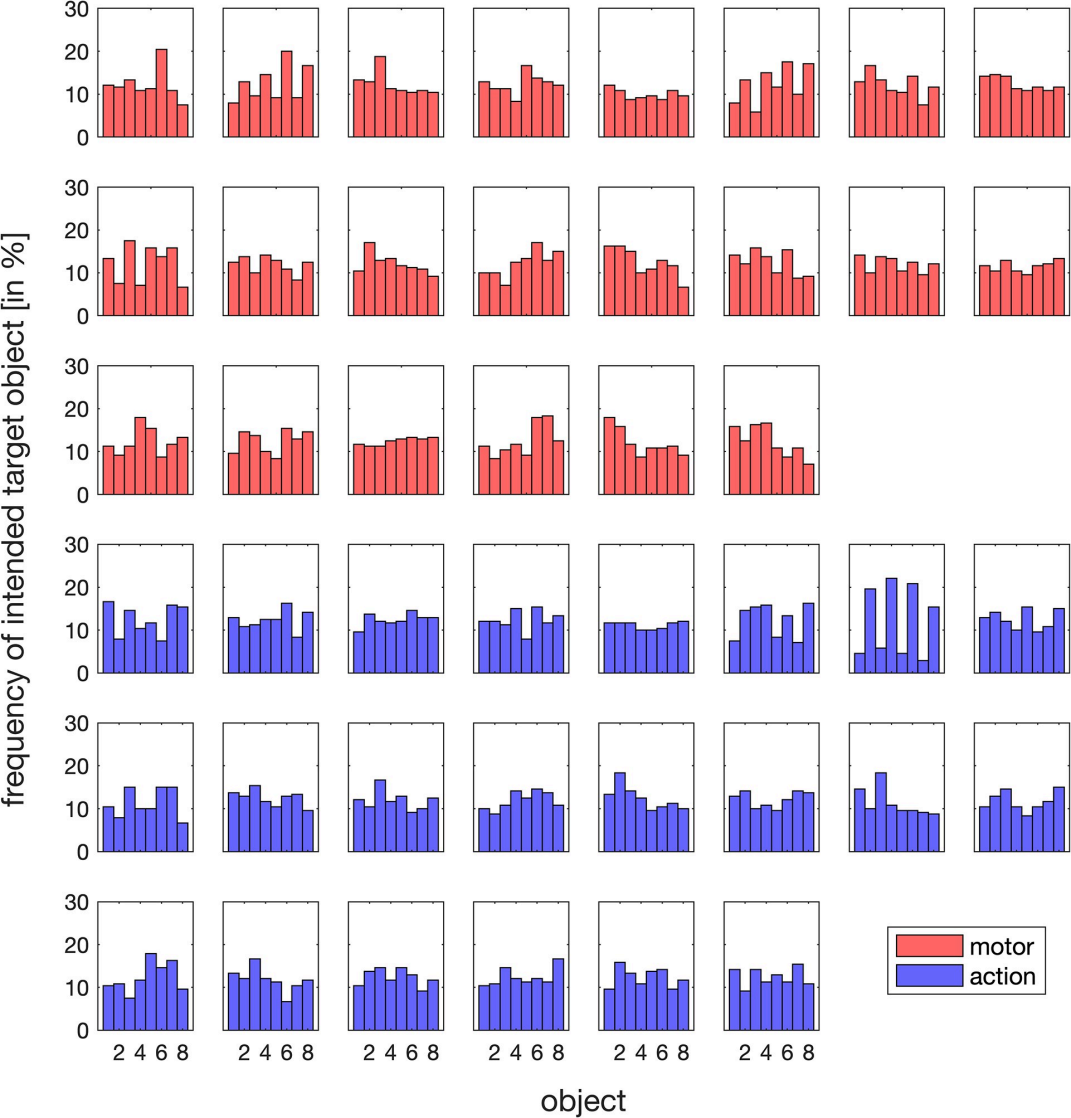
Relative frequency with which each participant chose each of the eight objects as intended target object throughout the entire experiment, depicted separately for motor (red) and action feedback (blue).

The stimuli were presented on a mid-grey background. At the beginning of each trial, a dark grey fixation cross (0.6 deg x 0.6 deg) was displayed at the centre of the screen. Before the trial continued, the participants’ eye position had to be within a fixation window (4 x 4 deg) that was centered around the fixation cross for at least 300 ms. Following detection of a stable initial fixation, eight objects appeared on a circular path around the fixation cross. The objects, clockwise and starting at 12 o’clock, were a star (light blue), a regular octagon (salmon), an isosceles triangle (purple), a rhombus (green), a square (dark blue), a regular hexagon (orange), a circle (turquoise) and a parallelogram (magenta). The centre of gravity of each object was aligned on an invisible circular path (7 deg radius). The star, the octagon, the rhombus, the hexagon and the circle had a diameter of 1.5 deg. The square had a side length of 1.5 deg, and the parallelogram and the triangle both had a base side of 1.5 deg and a height of 1.5 deg (Fig 1B). In any trial participants freely selected one of the objects as their intended target object. They were encouraged to use all objects over the course of the experiment. Participants followed the instruction and selected varying objects over the course of the experiment as intended target (Fig 2). Target areas were defined for each object. The target areas were circle segments of an invisible circle around the fixation cross (9 deg radius). Each target area extended 22.5 arc deg clockwise and counterclockwise around the respective object and extended from the fixation cross to the invisible circle line (exceeding the inner circular path by 2 deg). After the objects were drawn to the screen, participants continued to look at the fixation cross for another 500 to 1000 ms until the fixation cross was removed. If the fixation was interrupted before the offset of the fixation cross, a sinusoidal tone was played and the current trial was aborted and restarted. Subsequent events depended on the type of trial.

During circle-off trials, upon removal of the fixation cross, participants communicated their intended target object in the respective trial via an eye movement to the system. Once saccade onset was detected online, the objects on the circular path disappeared and thus no post-saccadic feedback was available. Participants then indicated the intended target object by pressing the corresponding key on the number pad of the keyboard. The correspondence between the intended target object and the corresponding key was established through the position of the objects on the circle and the position of the numbers on the number pad. For example, the number 6 on the number block corresponded to the purple triangle at 3 o’clock (Fig 1A).

During feedback trials, participants also communicated their intended target object in the respective trial via an eye movement to the system once the fixation cross was removed. As soon as saccade offset was detected online, the primary saccade end point was calculated. In both feedback conditions, a clockwise 45 arc deg rotation was added to the actual end point of the primary saccade to simulate a systematic error inherent to the system. The distortion remained constant throughout the experiment. This distorted saccade end point was used for feedback presentation and for decoding the intended target object. In the motor condition, participants received feedback about the end point of their saccade, distorted in the prescribed way, by a red dot (1 deg diameter) presented at the distorted saccade end point calculated by the system. In the action condition, participants received feedback about the object decoded by the system, including the same clockwise distortion, by coloring the decoded object red. In both conditions, participants indicated their intended target object by pressing the corresponding key on the number pad (Fig 1A). At least one second had to pass before participants could enter the intended object to make sure that they took the time to process the visual feedback. After the key press, the screen turned grey and a new trial started.

The decision about the decoded object was based on the distorted end point of the primary saccade. The object in whose associated target area the distorted end point fell was decoded as intended target object. Whether there was a decoding error was determined by means of a comparison between the intended target object (as indicated by keypress) and the decoded object (Fig 1B). If the distorted saccade end point did not fall in any of the target areas, e.g. because the saccade was too long and exceeded the invisible outer circle, the message “eye too far from any possible target” was displayed on the screen. This was done to encourage participants to perform more precise eye movements in the subsequent trials.

### Online detection of saccade onset and offset

During the circle-off trials, the objects disappeared once a primary saccade was initiated. Saccade onset was detected using an online criterion that was met if saccade velocity exceeded 100 deg/s and the eye had traveled at least 3 deg. During feedback trials, a feedback signal was presented as soon as saccade offset was detected online. The online criterion for saccade offset was met if the average eye velocity calculated over the last three samples fell below 30 deg/s. The saccade end point was defined as the last data point of the three samples used to calculate the eye velocity. In the motor condition, the median deviation of the saccade end point calculated with this criterion from the saccade end point calculated by the EyeLink software was 0.19 deg (*SD* = 0.05 deg). The median delay between offset of the primary saccade detected by the EyeLink software and onset of the post-saccadic feedback was 19 ms (*SD* = 1.89 ms). In the action condition, the median deviation of online detected saccade end point and saccade end point detected by the EyeLink software was 0.16 deg (*SD* = 0.07 deg). The median delay in the presentation of the post-saccadic feedback was 19 ms (*SD* = 3.28 ms). Thus, the visual feedback signal was provided well within the 200 ms following saccade offset during which visual sensitivity is increased [40,41] and changes in the visual scene can cause fast and implicit learning [20,42,43].

### Data analysis

All trials during which valid primary saccades were made were included in the analysis of primary saccade metrics and the assessment of the task performance. Primary saccades were considered valid if their amplitude was between 10.5 and 3.5 degrees of visual angle and if they had a duration of less than 100 ms. The saccade latency had to be over 100 ms. Following these inclusion criteria, 87.73% of the primary saccades and 87.68% of the object selections in the motor condition met the inclusion criteria. In the action condition, 86.14% of the primary saccades and 85.66% of the object selections were included in the data analysis. Secondary saccades following the primary saccade sometimes reflect error correction or attentional processes, but can also be made in response to feedback signals. Thus, we looked at secondary saccades with a latency between 100 and 500 ms following the primary saccade to cover a time window that was sufficiently large for also including secondary saccades that were made in response to feedback presentation. Further, we considered secondary saccades valid if their amplitude was smaller than 5 degrees of visual angle and their duration was less than 100 ms. These criteria applied to 38.69% of the secondary saccades in the motor condition and to 33.39% of the secondary saccades in the action condition. The data analysis was conducted in MATLAB (version R2022b) and R (version 4.3.1).

To test if participants adjusted their oculomotor behavior to a task goal using artificial feedback added to the post-saccadic scene, we investigated if the distorted saccade end point fell increasingly often within the target area of the intended object, i.e. we determined whether the probability of valid object decoding increased over time. We fitted a generalized linear mixed model with a binomial distribution (logit link) applying maximum likelihood estimation. The “glmmTMB” package [44] was used. First, we fitted a model including the predictors feedback type (factor with two levels: motor, action) and learning trial (continuous predictor). Further, we added a random intercept for each participant and an autoregressive correlation term for equally spaced time steps to account for the trial structure within the different feedback conditions and thus the dependence of successive data points (*AIC* = 6469.81). The continuous predictor trialnumber was centered to facilitate the interpretability of the results. This model was compared to a second model that featured a random slope such that the effect of trialnumber and feedback condition (as well as their interaction) could vary within each participant (*AIC* = 5734.32). Finally, in a third model, the predictor session order (factor with two levels: motor feedback first, motor feedback second) was added as a fixed effect to capture the influence of the order of the feedback conditions on the learning process (*AIC* = 5723.95). This model was selected as it had the lowest AIC value and was significantly superior to the next ranked model as indicated by the results of a chi-square difference test (*χ*^2^(4) = 18.37, *p* = .001). For the categorical factor feedback type, the factor level “motor” was selected as the reference category. For the categorical factor feedback order, the factor level “motor first” was selected as the reference category. Correspondingly, the intercept of the model corresponds to the probability of valid object decoding for motor feedback at learning trial 100, given that the feedback order is "motor feedback first". Using the “performance” package [45], we checked that no overdispersion, singularity or multicollinearity occurred. Further, we checked the normal distribution of the random effects. The predictive accuracy of the selected model was assessed using the Area Under the ROC curve (*AUC* = 83.38%), and the proportion of variance explained by both the fixed and random effects [46] was evaluated using the marginal (*R*^2^ = 0.186) and conditional *R*^2^ (*R*^2^ = 0.419). In addition, the “DHARMa”package [47] was used to check that the residuals followed the expected distribution.

For analyzing saccade curvature, saccade trajectories were first rotated such that each trajectory corresponded to a rightward saccade. This was necessary as primary saccades could have different directions due to the eight potential target objects on the circular path. After the rotation, the vertical position of the rotated saccade trajectory corresponds to the saccades’ deviation from a straight line between the start and end point. We then normalized the duration of the saccade and thus the length of the trajectory to 24 time steps using linear interpolation. After that, we calculated the area under the (rotated and normalized) saccade trajectory to quantify saccade curvature (for a review of the various methods for determining saccade curvature, see[48]).

Further, the effect of feedback type, learning phase and session order on primary saccade latency, primary saccade end point, curvature as well as angle between the eye position after the secondary saccade and the intended target was assessed using a mixed ANOVA. If the normality assumption was violated, we performed a permutation based mixed ANOVA [49] using the “permuco” package [50]. The p-value from the permutation-based approach (10000 permutations) is reported alongside the F-value from the parametric test. For post-hoc tests and other group comparisons, we performed t-tests. If the criteria for calculating parametric tests were not met, permutational t-tests were calculated (10000 permutations) using the “MKinfer” package [51]. The alpha-level was set to 0.05. To avoid inflation of the Type I error in case of multiple comparisons, the Bonferroni-Holm correction was applied. Testing for unimodality was done with Hartigan’s dip test [52] using the “diptest” package [53].

## Results

### Decoding error

In the current study, we aimed to investigate whether human participants are able to adjust their oculomotor behavior based on artificial feedback signals added to the post-saccadic image to achieve a task goal. The task assumes a fictional assistive device that decodes the intended target object and asks to communicate intention by eye movements such as to avoid decoding errors. Furthermore, we assess whether either feedback about the decoded object or feedback about the executed eye movement is better at eliciting adaptive changes to the oculomotor behavior to increase the frequency of valid object decoding.

**Fig 3:**
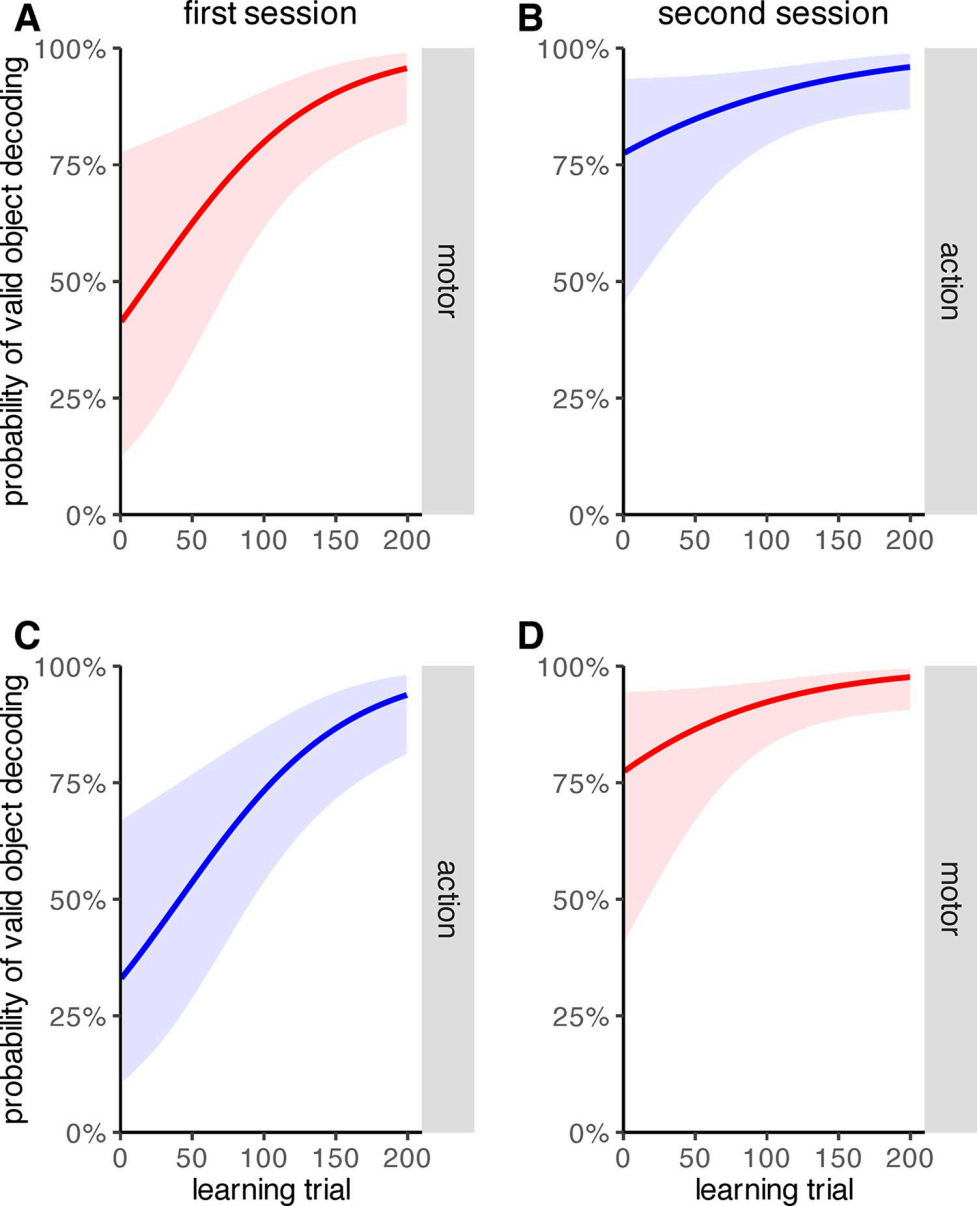
Probability of valid object decoding over the learning phase as predicted by the generalized linear mixed model, depicted separately for the different combinations of factor levels. A) Probability of valid object decoding in the motor condition when motor feedback was presented first. B) Probability of valid object decoding in the action condition when motor feedback was presented first. C) Probability of valid object decoding in the action condition when action feedback was presented first. D) Probability of valid object decoding in the motor condition when action feedback was presented first. Red represents the motor feedback condition, blue represents the action feedback condition. The solid lines indicate the model prediction, the shaded areas indicate confidence intervals.

We calculated a generalized linear mixed model to assess how learning trial, feedback type [motor feedback, action feedback] and feedback order [motor feedback first, action feedback first] affect the frequency of decoding errors. The parameter estimates are provided in Table 1. The model shows that the probability for valid target decoding increased significantly throughout the course of the experiment (*p* = .006). This was equally true for the motor and the action condition. Neither the feedback type influenced the probability of valid object decoding (*p* = .128), nor the interaction of feedback type and learning trial (*p* = .299), nor the interaction of feedback order and learning trial (*p* = .394). The influence of feedback order was significant (*p* = .016) as was the interaction of feedback order and feedback type (*p* < .001). This indicates a transfer of learning from one feedback type to the other, as performance in the second session started at a higher level than in the first session, irrespective of feedback type (Fig 3). In addition, the three-way interaction of feedback order, feedback type and learning trial (*p* = .031) was significant, suggesting that the interaction of learning trial and feedback type was different between the first and the second session (Fig 3).

**Table 1:** 

Parameter	*b*	*z*	*p*	95% CI
Intercept	1.38	2.98	.003**	[0.47, 2.29]
feedback type	0.83	1.52	.128	[-0.24, 1.90]
trial	0.02	2.76	.006**	[0.01, 0.03]
feedback order	1.11	2.41	.016*	[0.21, 2.02]
feedback type x trial	-0.01	-1.4	.299	[-0.02, 0.01]
feedback type x feedback order	-2.31	-4.93	< .001***	[-3.23, -1.39]
trial x feedback order	-0.005	-.85	.394	[-0.02, 0.01]
(feedback type x trial) x feedback order	0.01	2.16	.031*	[0.00, 0.02]

*Note*. Parameter estimates of the model.

The parameter estimates are provided as log odds.

### Primary saccades

**Fig 4:**
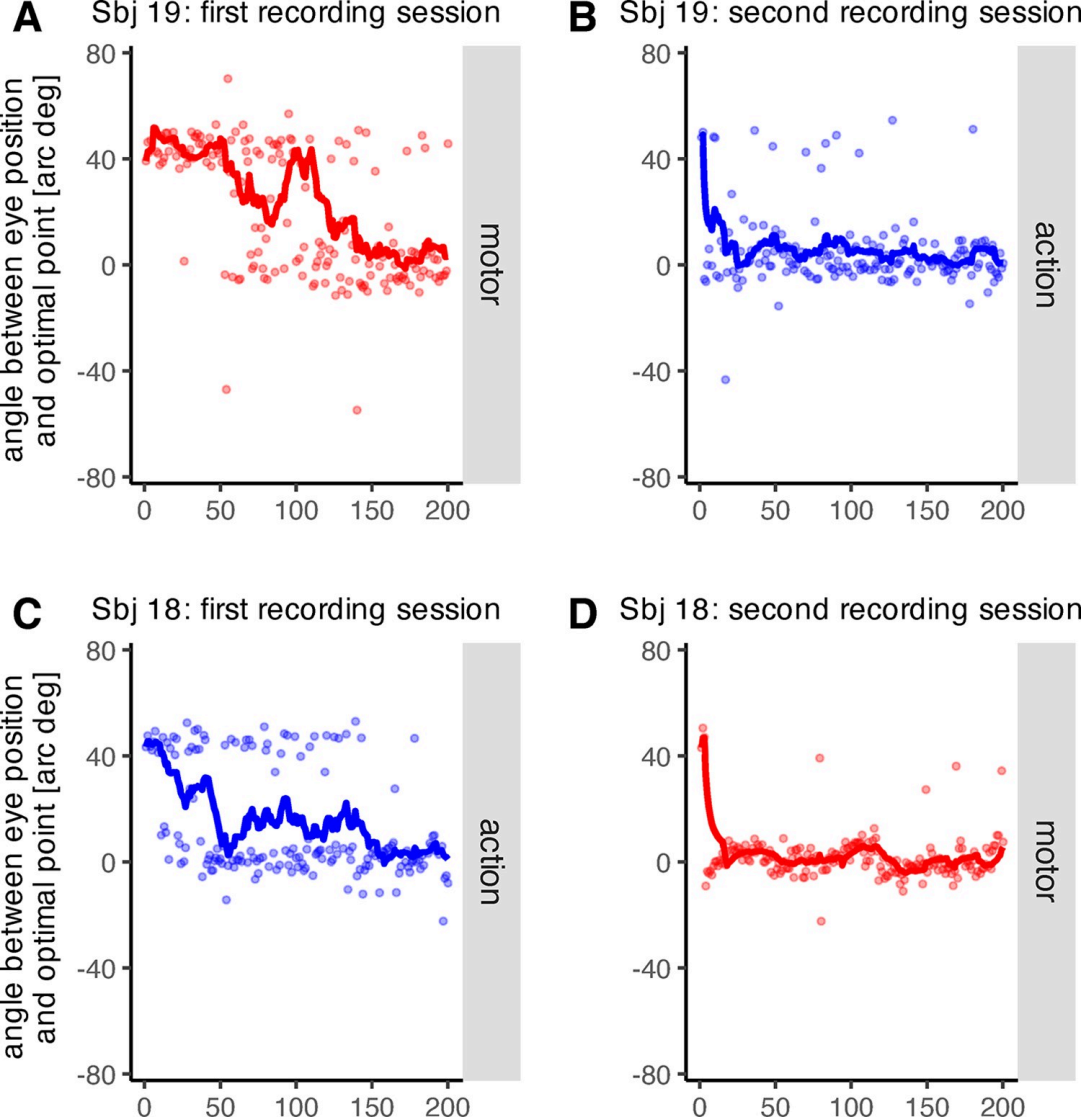
Example data from two participants. A) Participant 19: Angle between primary saccade end point and optimal point throughout the first recording session with motor feedback. B) Participant 19: Angle between primary saccade end point and optimal point throughout the second recording session with action feedback. C) Participant 18: Angle between primary saccade end point and optimal point throughout the first recording session with action feedback. D) Participant 18: Angle between primary saccade end point and optimal point throughout the second recording session with motor feedback.

The object decoding depended on the distorted primary saccade landing point. As the probability of valid object decoding increased over time, the distorted saccade landing point must have fallen increasingly often within the target area of the intended object. To achieve this, participants primary saccade end point must have counteracted the clockwise distortion that we imposed. We assessed the end point of the primary saccade with respect to two positions on the computer screen: the intended object and the optimal point. The optimal point corresponds to the position 45 arc deg counterclockwise from the intended target object. It is termed optimal point because directing the primary saccade to this position compensates for the full extent of the distortion, avoids a decoding error, and places the motor feedback about the saccade end point provided by the system directly at the position of the intended object. Over the course of the learning phase, participants made their saccades increasingly often toward the optimal point. Fig 4 shows two example participants. The comparison of the first and second recording session shows clear evidence of savings, i.e. the learning process from the first recording sessions facilitates the learning process during the second recording session. During the learning phase of the first recording session, participants initially made their saccades toward the intended target object. The feedback indicated decoding errors and participants consequently explored new saccade vectors that could counteract the systematic distortion. Once the participants had identified the oculomotor behavior that maximized the probability of task success, they made the saccades increasingly often toward the optimal point. During the learning phase of the second recording session, participants quickly reapplied the oculomotor behavior learned during the previous session once they were presented with the new feedback type. Thus, the frequency with which saccades were made toward the intended target decreased much more quickly during the second than during the first recording session.

**Fig 5:**
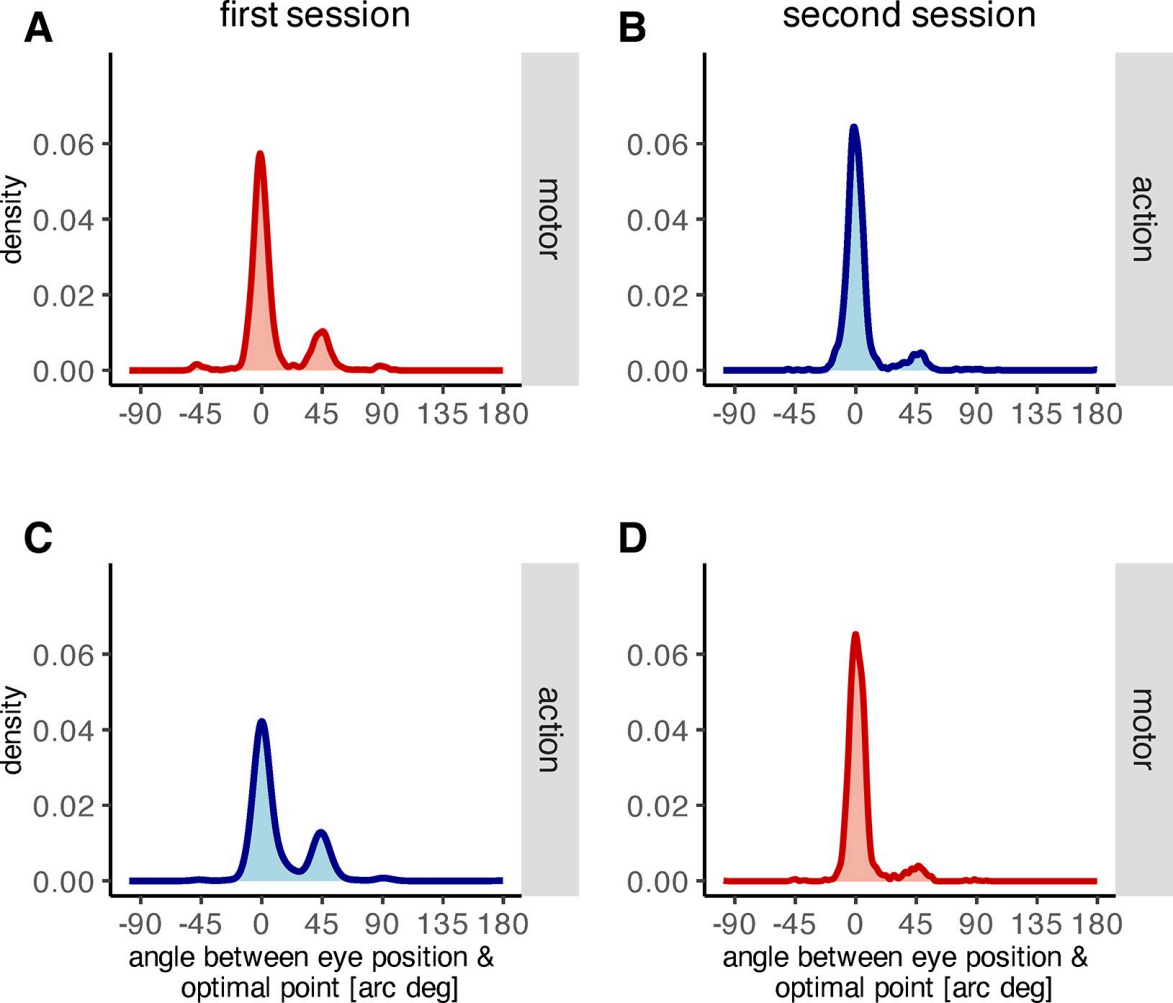
Density plot of the angles between primary saccade end points and optimal point A) throughout the first recording session with motor feedback, B) throughout the second recording session with action feedback, C) throughout the first recording session with action feedback, D) throughout the second recording session with motor feedback.

Correspondingly, the distribution of the angle between the saccade landing point and the optimal point was bimodal during the learning phase of the first recording session, both for motor (Fig 5A, *D* = 0.032, *p* < .001, Hartigan’s dip test) and action feedback (Fig 5C, *D* = 0.041, *p* < .001, Hartigan’s dip test). During the learning phase of the second recording session however, when participants quickly reapplied the previously learned behavior, the deviation from a unimodal distribution was no longer significant, neither for motor (Fig 5C, *D* = 0.008, *p* = .529, Hartigan’s dip test) nor for action feedback (Fig 5D, *D* = 0.012, *p* = .070, Hartigan’s dip test). These results provide further evidence for savings from the first to the second recording session. More specifically, savings only occurred during the learning procedure of the second recording session, i.e., after the feedback signal was added to the post-saccadic scene. The average angle between primary saccade end point and intended target object did not deviate significantly from zero during the pre-learning circle-off trials of the second recording session, neither when action feedback had been presented during the first recording session (*t*(10) = 0.25, *p* = .810, two-sided t-test) nor when motor feedback had been presented during the first recording session (*p*_*perm*_ = .178, two-sided permutation t-test). These results indicate that the primary saccades, made during the pre-learning circle-off trials of the second recording session, were made toward the intended target object and were unaffected by the learning process of first recording session.

**Fig 6:**
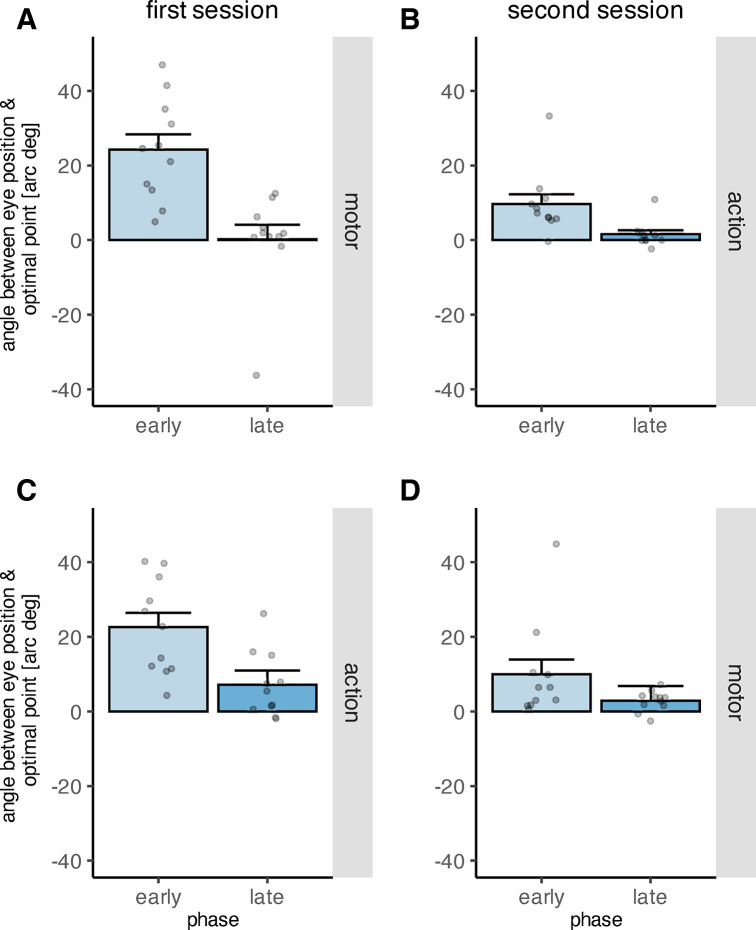
Mean angle between primary saccade end point and optimal point measured during the early and late learning phase, depicted separately for the different combinations of factor levels. The grey dots indicate individual data points. A) Mean angle between primary saccade end point and optimal point in the motor condition when motor feedback was presented first. B) Mean angle between primary saccade end point and optimal point in the action condition when motor feedback was presented first. C) Mean angle between primary saccade end point and optimal point in the action condition when action feedback was presented first. D) Mean angle between primary saccade end point in the motor condition when action feedback was presented first. The error bars indicate the standard error of the mean.

We analyzed the data across participants by assessing the effects of phase, feedback type and feedback order on the angle between the actual saccade end point and the optimal point systematically in a mixed ANOVA. The results showed a significant main effect of phase [early learning, late learning] (*F*(1,20) = 54.18, *p*_*perm*_ < .001) on the angle between primary saccade end point and the optimal point (Fig 6). The main effect of feedback type (*F*(1,20) = 0.26, *p*_*perm*_ = .615) was not significant, indicating that both feedback types allowed to counteract the distortion to achieve their task goal, i.e. to have the system decode the intended object. The main effect of feedback order was also not significant (*F*(1,20) = 0.31, *p*_*perm*_ = .580). However, the interaction of feedback order and feedback type was significant (*F*(1,20) = 17.12, *p*_*perm*_ = .001), as was the three-way interaction between phase, feedback type and feedback order (*F*(1,20) = 11.24, *p*_*perm*_ = .004). The interactions between phase and feedback order (*F*(1,20) = 1.68, *p*_*perm*_ = .206) and between phase and feedback type (*F*(1,20) = 1.10, *p*_*perm*_ = .296) were not significant. The mean angle between primary saccade end point and optimal point decreased from the early to the late learning phase (*p*_*perm*_ < .001; one-sided permutation t-test). Although the angle between primary saccade end point and the optimal point decreased significantly during learning, the adjustment of the primary saccade was not complete when aggregated over both feedback conditions (*p*_*perm*_ = .023, one-sided permutation t- test). However, when looking at the adjustment of the primary saccade end point separately for motor and action feedback, the adjustment to the distortion was complete for motor feedback (*p*_*perm*_ = .229, one-sided permutation t- test), but not for action feedback (*p*_*perm*_ = .011, one-sided permutation t- test). Post-hoc tests for the interaction between feedback type and feedback order demonstrated that when action feedback was presented first, the angle between the primary saccade end point and optimal point was larger during the first recording session (during which action feedback was presented) than during the second recording session during which motor feedback was presented (*p*_*perm*_ = .007, one-sided permutation t- test), confirming that learning transferred from the first to the second session. However, when motor feedback was presented first, the angle between primary saccade end point and optimal point was not significantly larger for motor than for action feedback (*p*_*perm*_ = .083, one-sided permutation t- test). No difference regarding the magnitude of the angle occurred when the respective first and second recording session of the two groups of participants (motor feedback first vs action feedback first) were compared (all *p*_*perm*_ > .01, two-sided permutation t-tests). The average angle between primary saccade end point and optimal point during the early and late learning phase can be found in Table 2 (separately for the levels of the categorical factors feedback type and feedback order).

**Table 2:** Average angle between primary saccade end point and optimal point.

feedback order		motor feedback first				action feedback first			
feedback type		motor		action		motor		action	
		*M*	*SD*	*M*	*SD*	*M*	*SD*	*M*	*SD*
phase	early	24.27	13.54	9.70	8.63	9.98	13.00	22.59	12.75
	late	0.21	12.90	1.60	3.37	2.89	2.76	7.15	8.76

*M* and *SD* [in arc deg] represent mean and standard deviation, respectively.

**Fig 7:**
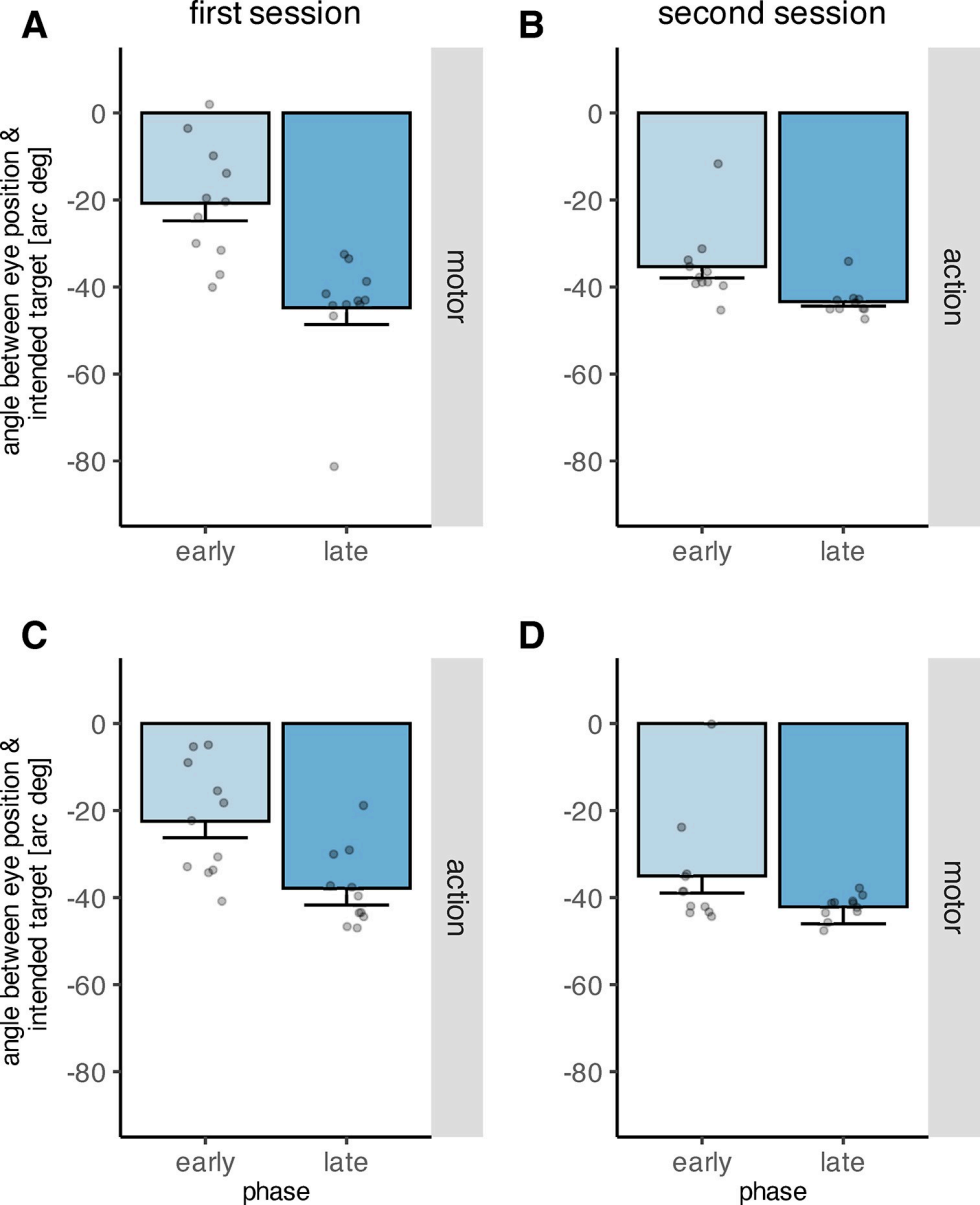
Mean angle between primary saccade end point and intended target object measured during the early and late learning phase, depicted separately for the different combinations of factor levels. The grey dots indicate individual data points. A) Mean angle between primary saccade end point and intended target in the motor condition when motor feedback was presented first. B) Mean angle between primary saccade end point and intended target in the action condition when motor feedback was presented first. C) Mean angle between primary saccade end point and intended target in the action condition when action feedback was presented first. D) Mean angle between primary saccade end point and intended target in the motor condition when action feedback was presented first. The error bars indicate the standard error of the mean.

Correspondingly, the effect of phase on the angle between primary saccade end point and intended object was significant (*F*(1,20) = 54.18, *p*_*perm*_ < .001), while neither the main effect of feedback type (*F*(1,20) = 0.26, *p*_*perm*_ = .615) nor the main effect of feedback order were significant (*F*(1,20) = 0.31, *p*_*perm*_ = .580). The interactions between phase and feedback order (*F*(1,20) = 1.67, *p*_*perm*_ = .206) and between phase and feedback type (*F*(1,20) = 1.10, *p*_*perm*_ = .296) were not significant. However, the interactions between feedback type and feedback order (*F*(1,20) = 17.12, *p*_*perm*_ = .001) and between phase, feedback type and feedback order (*F*(1,20) = 11.24, *p*_*perm*_ = .004) were significant. In addition, the angle between primary saccade end point and intended object increased from early to late learning procedure (*p*_*perm*_ < 0.001, one-sided permutation t-test), confirming that the participants learned to counteract the distortion by aiming their primary saccades toward the optimal point rather than the intended object (Fig 7).

**Fig 8:**
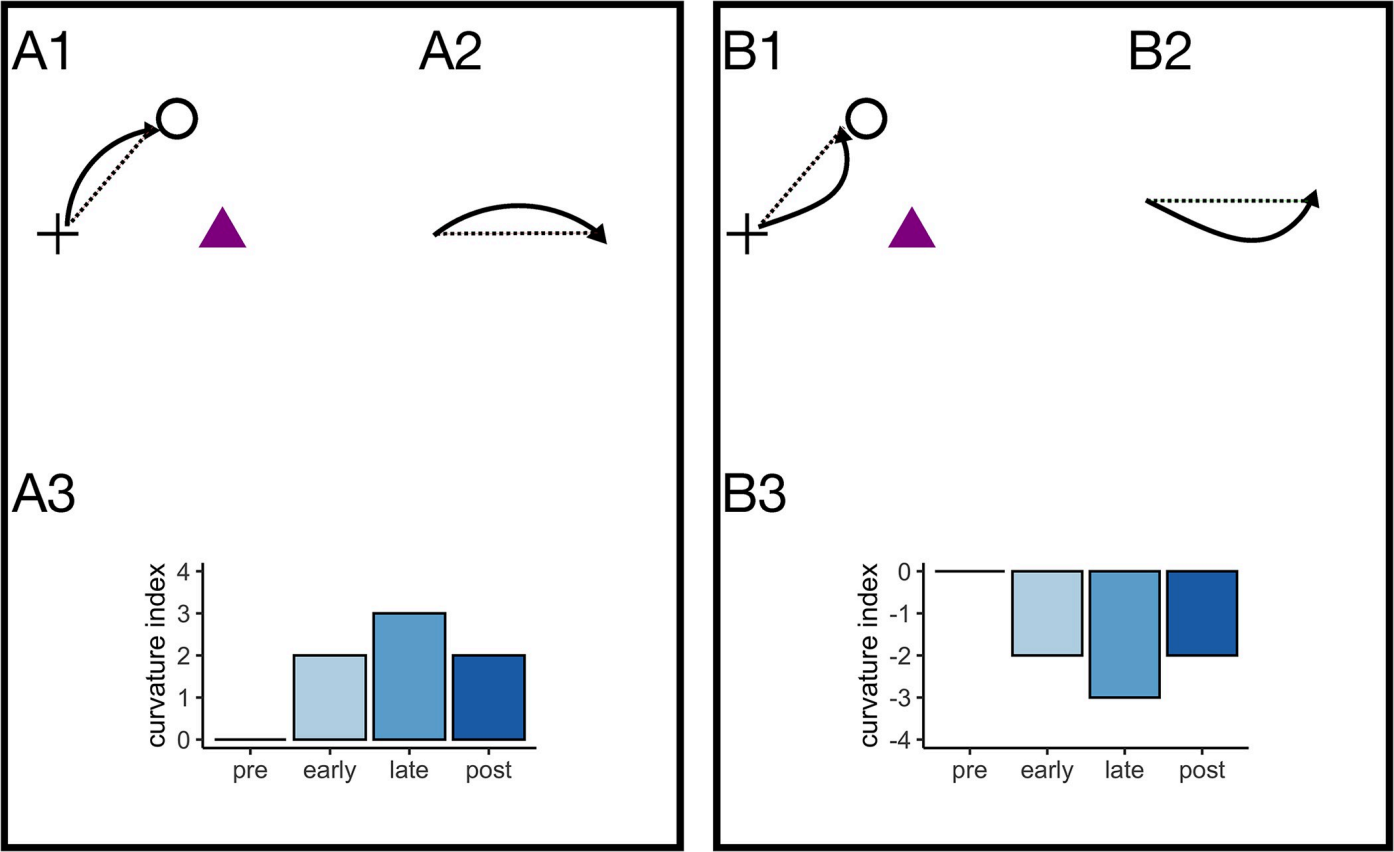
Expected saccade trajectories (solid lines) and curvature indices in case of a distractor effect (A) and forward model adaptation (B). To select the intended object (purple triangle), the saccade is made toward the optimal point (transparent circle). A1) The saccade trajectory curves away from the intended target which competes with the optimal point. A2) The saccade trajectory deviates from a straight line (dashed line) between saccade start point and saccade end point in counterclockwise direction. This deviation is quantified as the area between the dashed line and the saccade trajectory. A3) During pre-learning, before the feedback is introduced, saccades are made toward the intended target object. No adjustment of the oculomotor behavior has occurred yet and no systematic effect on curvature is expected. During early learning, the feedback is introduced and participants begin to make their saccade toward the optimal point. The distractor effects develops and reaches its maximum during late learning. When the feedback is removed, the strength of the distractor effect decreases. B1) The saccade trajectory initially goes toward the intended object, but later curves in direction of the optimal point. B2) The saccade trajectory deviates from a straight line in clockwise direction. A3) During pre-learning, no adaptation has occurred yet and no systematic effect is expected. During early learning, adaptation of the forward model develops and is strongest during late learning. When the feedback is removed, adaptation magnitude decreases and curvature decreases correspondingly.

In the current study, eight objects were presented simultaneously and the participants selected one of them as their intended target object in each trial. Because of the distortion that was added to the saccade end point, participants had to adjust their oculomotor behavior to the rotation and make their saccade toward the optimal point instead of the intended object. Performing any goal-directed movements in a scenario that involves multiple possible interaction objects or goal locations requires that competition between objects or locations in the visual scene is resolved. In this view, making a saccade toward the optimal point requires that the optimal point is selected as goal location, and that the optimal point wins the competition over the intended target object. This would render the intended object a distractor. To test this hypothesis, we analyzed saccade curvature. Saccade trajectories can deviate toward distractor items or away from them. It is assumed that deviation toward a distractor item reflects unresolved competition between objects at saccade onset, while deviation away from a distractor indicates that top-down preparation has influenced the target selection and that the competing distractor location is inhibited. Congruently, deviation toward distractors often occurs for short saccade latencies, while deviation away from distractors occurs for longer latencies [48,54,55]. In addition, saccade adaptation in response to intra-saccadic target displacement involves the adaptation of a forward model, which is typically reflected by an increase in saccade curvature, especially toward the end of the saccadic eye movement [56] (Fig 8). In our analysis, deviation from a straight line between saccade start point and saccade end point in clockwise direction is indicated with a negative sign, deviation from a straight line in counterclockwise direction is indicated with a positive sign. Thus, in trials during which the intended target is decoded correctly, a positive deviation indicates curvature away from the intended target, congruent with a distractor effect. A negative curvature index indicates either that the trajectory is curved toward the intended target, or that the saccade first goes for the intended target and then is corrected toward the optimal point (congruent with adaptation of the forward model). The respective saccade trajectories and expected curvature indices for both scenarios are illustrated in Fig 8.

**Fig 9:**
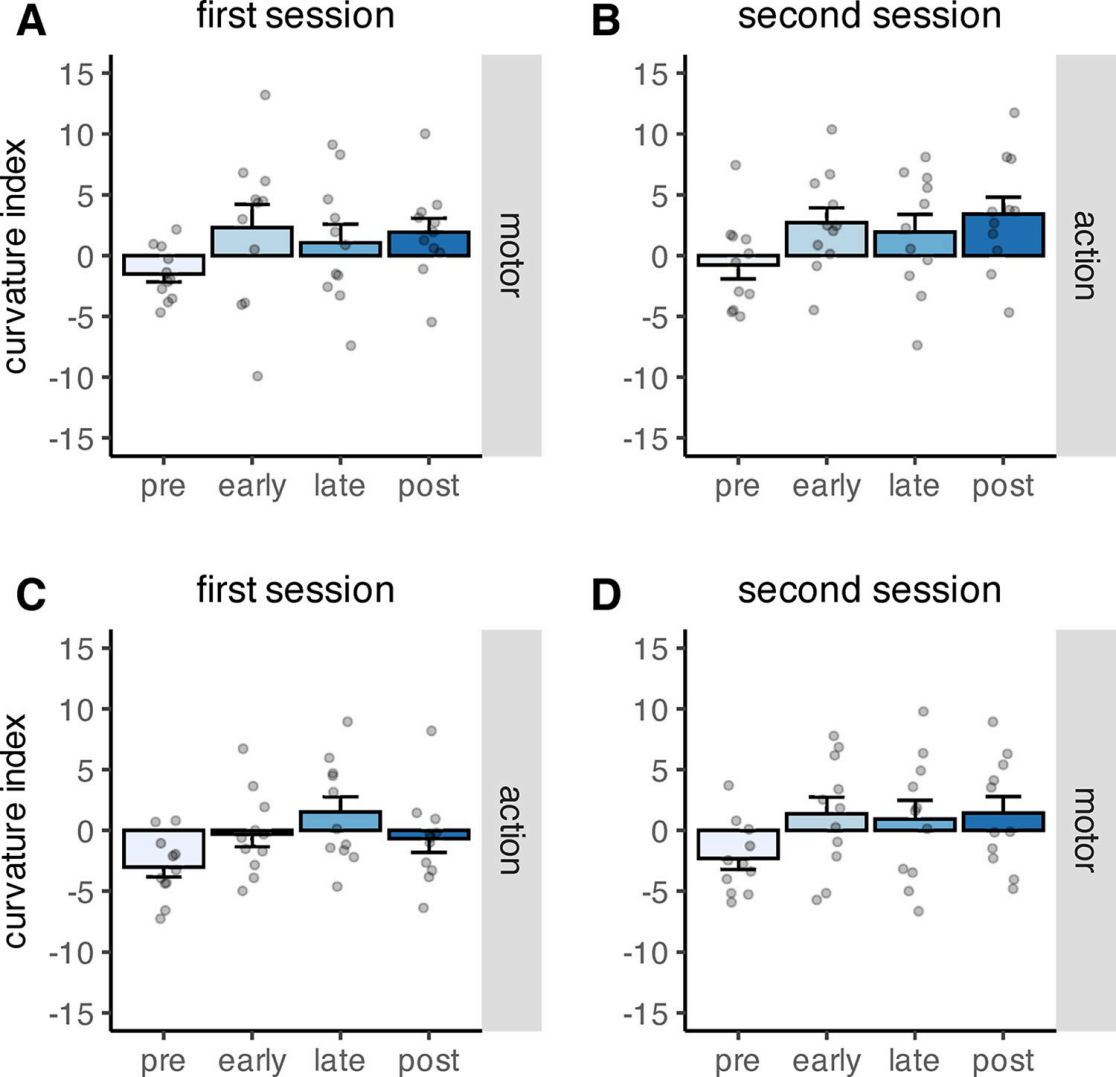
Mean curvature index during the pre-, early-, late- and post- learning phase, depicted separately for the different combinations of factor levels. The grey dots indicate individual data points. A) Mean curvature index in the motor condition when motor feedback was presented first. B) Mean curvature index in the action condition when motor feedback was presented first. C) Mean curvature index in the action condition when action feedback was presented first. D) Mean curvature index in the motor condition when action feedback was presented first. The error bars indicate the standard error of the mean.

We assessed the effects of learning phase, feedback type and feedback order on saccade curvature. Only learning phase had a significant effect on saccade curvature (*F*(3,60) = 10.12, *p* < .001). The main effects of feedback order (*F*(1,20) = 1.59, *p* = .222) and feedback type (*F*(1,20) = 0.01, *p* = .928) were not significant, as well as their interaction (*F*(1,20) = 3.35, *p* = .082). Neither the interaction between phase and feedback type (*F*(2.11, 42.11) = 0.37, *p* = .706), nor between phase and feedback order (*F*(3,60) = 0.69, *p* = .560), nor the three-way interaction between feedback order, phase and feedback type were significant (*F*(2.11, 42.11) = 0.51, *p* = .615). Curvature during the pre-learning phase was significantly different from early- (*p*_*perm*_ < .001, two-sided permutation t-test), late- (*p*_*perm*_ < .001, two-sided permutation t-test) and post-learning (*p*_*perm*_ < .001, two-sided permutation t-test). The other comparisons were all not significant (all *p*_*perm*_ > .800). The pattern of results indicates that the saccade trajectory deviated away from the intended object, congruent with a distractor effect on saccade curvature (Fig 9).

**Fig 10:**
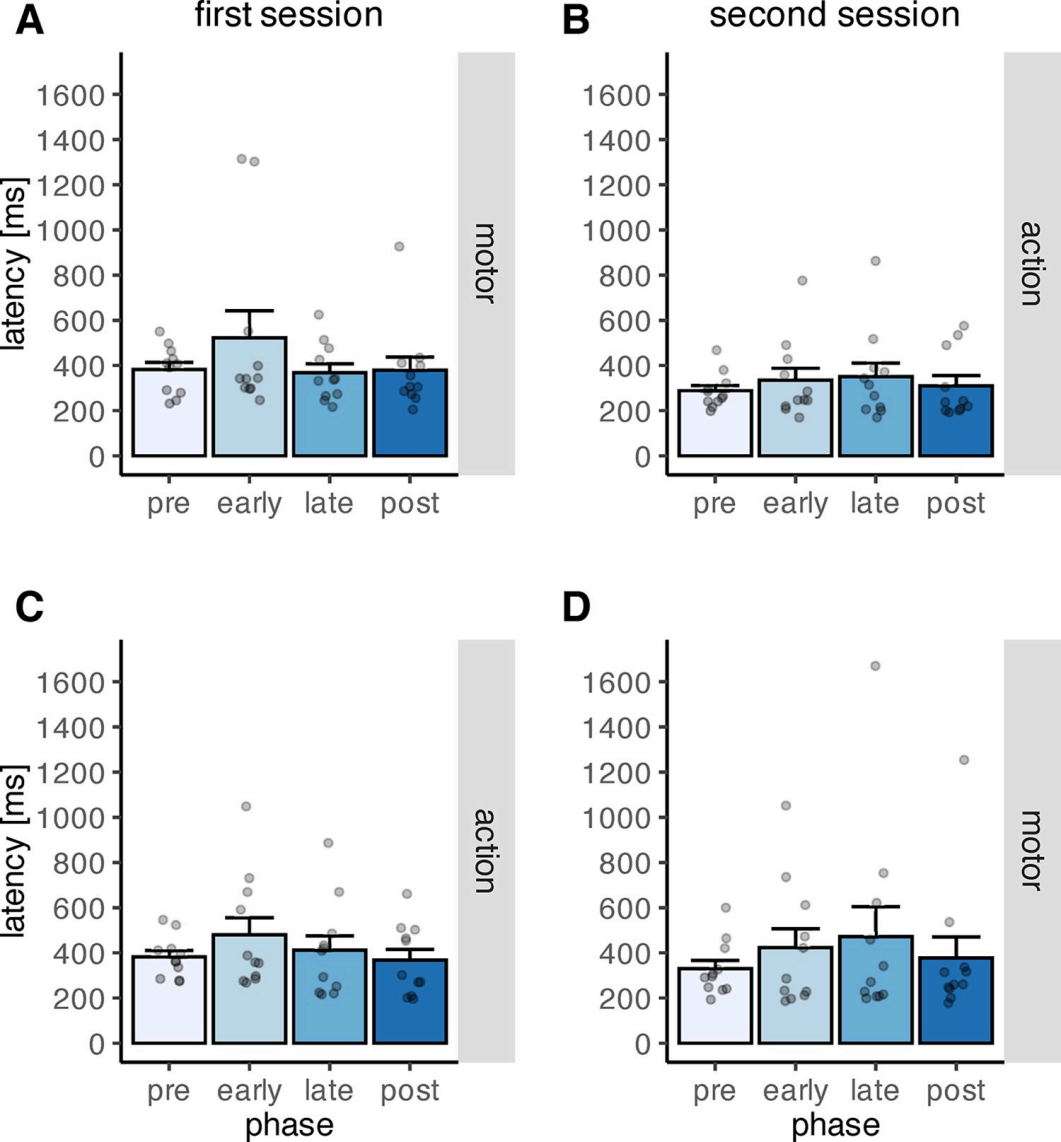
Primary saccade latency during the four phases “pre-learning”, “early learning”, “late learning” and “post-learning”, depicted separately for the different combinations of factor levels. The grey dots indicate individual data points. A) Primary saccade latency in the motor condition when motor feedback was presented first. B) Primary saccade latency in the action condition when action feedback was presented first. C) Primary saccade latency in the action condition when action feedback was presented first. D) Primary saccade latency in the motor condition when action feedback was presented first. The error bars indicate the standard error of the mean.

To assess if feedback type, feedback order, or phase affected the latency of the primary saccades, a mixed ANOVA was performed. The influence of phase [pre learning, early learning, late learning, post learning] (*F*(3,60) = 2.99, *p*_*perm*_ = .038) was significant, while neither the main effects of feedback type [motor, action] (*F*(1,20) = 1.83, *p*_*perm*_ = .190) nor feedback order (*F*(1,20) = 0.25, *p*_*perm*_ = .628) were significant. The interactions between phase and feedback order (*F*(3,60) = 0.34, *p*_*perm*_ = .797), between feedback type and feedback order (*F*(20,1) = 2.80, *p*_*perm*_ = .108), and between phase and feedback type (*F*(3,60) = 0.27, *p*_*perm*_ = .851) were not significant. However, the three-way interaction of phase, feedback type and feedback order was significant (*F*(3,60) = 3.09, *p*_*perm*_ = .031), indicating that the interaction of phase and feedback type was influenced by feedback order (Fig 10). Pairwise comparisons between the different phases showed that primary saccade latency was significantly shorter during pre-learning than during early learning, indicating that participants took more time to initiate their saccades when the feedback was initially introduced (*p*_*perm*_ = .049, two-sided permutation t-test). Further, primary saccade latency was longer during early learning than after the learning procedure (*p*_*perm*_ = .049, two-sided permutation t-test), indicating that it took participants longer to initiate a saccade when the feedback was first introduced compared to after the learning procedure. The other comparisons were not significant (all *p*_*perm*_ > .01). The average saccade latency during the different learning phases is depicted in Fig 10, separately for both feedback types and for both levels of the factor feedback order. Means and standard deviations are reported in Table 3.

**Table 3:** Average saccade latency.

feedback order		motor feedback first				action feedback first			
feedback type		motor		action		motor		action	
		*M*	*SD*	*M*	*SD*	*M*	*SD*	*M*	*SD*
phase	pre	381.59	105.80	288.17	78.15	330.37	120.24	381.94	91.73
	early	521.89	396.54	334.41	175.89	422.35	278.25	479.47	251.02
	late	368.37	127.33	350.74	198.61	471.37	439.62	411.24	211.35
	post	378.10	195.17	309.87	148.06	377.83	305.83	368.05	157.04

*M* and *SD* [in ms] represent mean and standard deviation, respectively.

### Secondary saccades

**Fig 11:**
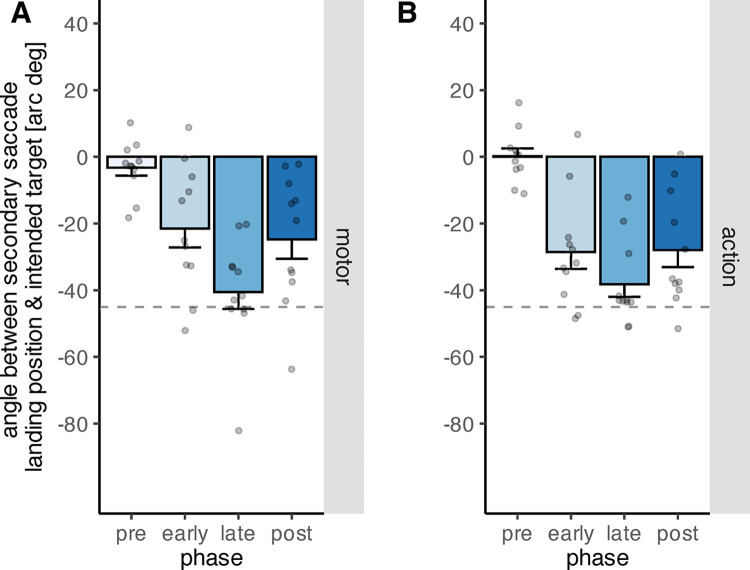
Angle between the eye position following secondary saccades and the intended target object during the four phases “pre-learning”, “early learning”, “late learning” and “post-learning”, depicted separately for motor (A) and action (B) feedback. The grey dots indicate individual data points. The dashed line indicates the angular position of the optimal point. The error bars indicate the standard error of the mean.

To assess how adjusting the oculomotor behavior to task demands on the basis of artificial feedback signals affects secondary saccades, we analyzed the angle between the eye position following the secondary saccades and the intended object (Fig 11). Not all participants made valid secondary saccades during the circle-off trials before and after the learning procedure, because the object array was removed upon saccade onset and no visual feedback was available after saccade landing. Thus, we removed eleven participants from this analysis who had missing data during either the pre- or post-learning phase. As a consequence, we only assessed the effect of phase and feedback type on the angle, and refrained from including the factor variable order as the data for this analysis were no longer balanced due to excluding participants. The main effect of phase was significant (*F*(3,30) = 23.59, *p* < .001), while the main effect of feedback type was not (*F*(1,10) = 0.09, *p* = .766). The interaction between phase and feedback type was not significant(*F*(3,30) = 1.17, *p* = .338). The angle between the eye position following the secondary saccade and the intended target did not differ significantly from zero during pre-learning neither during the motor feedback (*t*(10) = -1.351, *p* = .206, two-sided t-test), nor the action feedback condition (*t*(10) = 0.079, *p* = .939, two-sided t-test). However, the angle increased from pre-learning to early learning (*p*_*perm*_ < .001, two-sided permutation t-test), and increased further from early learning to late learning (*p*_*perm*_ = .013, two-sided permutation t-test), indicating that the eye position following the secondary saccade approached the optimal point. Correspondingly, the increase from pre- to late learning is also significant (*p*_*perm*_ < .001, two-sided permutation t-test), and so is the increase from pre- to post-learning (*p*_*perm*_ < .001, two-sided permutation t-test). From late learning to post-learning however, the angle decreased (*p*_*perm*_ = .016, two-sided permutation t-test) and the difference between early- and post-learning was not significant (*p*_*perm*_ = .688, two-sided permutation t-test). Means and standard deviations are reported in Table 4.

**Table 4:** Average angle between eye position following secondary saccade and intended object.

		feedback type					
		motor			action		
phase		*M*		*SD*	*M*		*SD*
pre		-3.26		8.00	0.19		7.81
early		-21.48		19.00	-28.56		16.65
late		-40.55		16.68	-38.19		12.59
post		-24.73		19.37	-27.97		17.02

*M* and *SD* [in arc deg] represent mean and standard deviation, respectively.

## Discussion

We investigated if human participants use artificial feedback signals added to the post-saccadic scene to adjust their oculomotor behavior to a task goal. Participants selected an object from a set of objects and had to indicate this intended target object via their gaze to a simulated assistive system. A systematic distortion, a clockwise 45 arc deg rotation, was added to the primary saccade end point to simulate decoding errors that may originate not only from the oculomotor behavior of the user but also from the system. This distorted primary saccade end point determined at which location the post-saccadic feedback signal would be presented. The post-saccadic feedback provided either information about the intended object as decoded by the system, or it provided information about the decoded end point of the saccade. Therefore, the visually presented feedback signal was not a veridical representation of the executed eye movement, and decoding errors occurred because of the added distortion. To achieve the task goal, i.e. to have the system correctly decode the intended object, participants had to aim their primary saccades toward the optimal point rather than the intended object such that the distorted saccade landing point fell in the target area of the intended object.

We found that the probability of valid object decoding increased over time, i.e. participants adjusted their oculomotor behavior on the basis of artificial feedback signals until they succeeded in the task. In addition, the probability of valid object decoding was lower at the beginning of the learning procedure of the first than at the beginning of the learning procedure of the second recording session, indicating that a transfer of learning occurred from the first to the second recording session, even though two different feedback types were used. This transfer of learning from one recording session to the next resembles savings. Savings are a characteristic of motor learning in general [57], and they also have been demonstrated following saccade adaptation [58]. Savings describe that prior learning can speed up subsequent relearning of the same behavior even after the behavioral manifestation of prior learning has been extinguished. In our study, the magnitude and direction of the imposed distortion was the same in both feedback sessions. It is likely that memory of the oculomotor behavior that led to the achievement of the task goal in the first recording session (aiming their saccade toward the object 45 arc deg counterclockwise to the intended target) facilitated the learning process in the second recording session, although the feedback signal driving the adaptation process was a different one. These findings indicate that participants in our study were capable of generalizing the memory of a previous oculomotor learning process to a new scenario several days later. This result is particularly encouraging considering that potential users of an assistive device controlled by a human-machine interface will not master effortless communication with the device on the first try, but will likely require training over an extended period of time and in different scenarios.

The average angle between primary saccade end point and optimal point decreased from early to late learning phase. Participants learned to counteract the distortion by making their saccades increasingly often toward the optimal point. Thus, artificial feedback added to the post-saccadic scene is a suitable means to induce adaptive changes to the oculomotor behavior. Participants in our study were told that the system could make errors. Therefore, participants likely did not attribute the errors internally, for example to erroneous motor performance, but externally. Still, the oculomotor behavior was adjusted to the task. The adaptive changes did not decrease the distance between intended target and fovea as in conventional saccade adaptation, but aimed at improving task performance.

One of our main questions was if action feedback or motor feedback were better suited to elicit learning and the corresponding adaptive changes that were necessary to succeed in the task. We found that the feedback type neither affected the overall task performance, as measured by decoding error, nor the rate at which learning occurred. Correspondingly, the angle between primary saccade end point and optimal point decreased following the presentation of both feedback types in a similar manner, i.e. the participants made their saccade closer to the optimal point than the intended target object. However, the adjustment to the distortion was only complete for motor feedback but not for action feedback. This is probably due to the different nature of the two feedback types: the motor feedback on one hand provided information about the executed movement itself and further about the magnitude and direction of the error. The action feedback on the other hand provided information about the decoded target object and therefore unambiguous feedback about task success or failure. When motor feedback was presented, the participants probably tried to minimize the post-saccadic error signal by placing the red dot as close as possible to the intended target or even on top of it. This required that the primary saccade was aimed precisely at the optimal point. When action feedback was presented, the intended target was colored when the distorted saccade landing point fell in the target area around the intended target, not just when the saccade was made precisely toward the optimal point. Thus, a broader range of saccade landing points led to task success.

Oculomotor learning in response to artificially induced errors [20,59] is just one example of motor learning. The motor system in general adapts to perturbations, for example in the context of reaching movements [32,34,35,60–63]. Manipulations of visual feedback [63] or mechanic distortions of limb movements [35] can induce error-reducing adaptive changes. Further, motor commands can be adapted based both on sensory error feedback and based on reward feedback [32,33]. In both cases, adaptation of the motor command is a gradual process that can lead to aftereffects [32,34,35]. It is suggested that adaptation following error-based learning develops because an internal model is updated, i.e. the mapping of a motor command and its consequences is recalibrated [64,65]. For reward-based adaptation, it is suggested that adaptation develops by sampling motor commands until identifying the one that maximizes the probability of task success [66]. Foveating a target object is rewarding in itself [67–70]. Paeye et al. [70] demonstrated that saccade vectors that were more likely to be followed by the foveal presentation of a target were executed more frequently than saccade vectors that were less likely to be followed by the presentation of the target stimulus. Thus, the oculomotor behavior was adjusted to the regularities of the search display to increase the probability of finding the target and thus to succeed in the current task. In our current study, eye movement behavior was also adjusted to the regularities of the environment (here: the systematic distortion inherent to the simulated AI system) to increase the probability of valid object decoding and thus to succeed in the task: once the counterclockwise rotation to the saccade vector that maximized the probability of task success was identified, it was executed more frequently. Some studies indicate that error-based and reward-based learning can operate jointly to find the optimal motor behavior for the task [32,33,71]. This is also possible in the current study. The artificial feedback signal added to the post-saccadic scene could have been used for the evaluation of a sensory error and the evaluation of a reward-based error simultaneously. Because the artificial feedback signal does not reflect the actual, but the distorted saccade landing position, the post-saccadic feedback signal initially appears at an unexpected location. The mismatch between the expected and actual position of the feedback signal was reduced through adaptive changes to the oculomotor behavior. Further, the feedback signal was an indicator of task performance, and participants adjusted their oculomotor behavior such that the probability of task success was maximized. Possibly, error-based learning was involved to a larger extent in the learning process following presentation of motor feedback, because precise error information regarding magnitude and direction was provided. Further, learning from action feedback might rely more heavily on reward-based learning, because 1) direct information about the success of the preceding motor action was given and 2) information about the motor error was less precise. This would be in line with results suggesting that the involvement of reward-based learning depended on presence and quality of the sensory error feedback [32,33]. We hypothesize that sensory error-based and reward-based learning interacted in the current study. Conventional saccade adaptation, as well as other adaptive processes, often rely on multiple mechanisms. For example, contribution of implicit and explicit mechanisms has been debated [28,72].While the traditional view on conventional saccadic adaptation is that adaptive adjustments of the saccade occur implicitly and reflect neural reorganization caused by a systematic post-saccadic error, we suggest that tasks or situational demands are just as relevant in the context of saccade adaptation. Those top-down influences define the error signal driving adaptive changes, which becomes especially critical outside the laboratory. In natural viewing, there is no error-inducing intra-saccadic displacement of objects. Instead, the post-saccadic error signal in case of an over- or undershooting saccade is likely to indicate whether 1) the saccade was suitable to provide clear vision of the target object or 2) whether the visual information is suitable to inform and guide upcoming actions. This hypothesis is backed by research demonstrating that clear vision of the saccade target is rewarding [67–70] and that saccade adaptation occurs to improve task performance [26,28,29]. If the executed saccade was not suitable to provide clear vision of the target or to guide upcoming actions, the oculomotor behavior should be modified and, possibly, neural reorganization might develop. To investigate differences and similarities between conventional adaptation and adaptation driven by task errors, well known features of conventional saccade adaptation like gradual development, persisting aftereffects [42,43,73] and also a shift of the perceived location of objects in adaptation direction [74–76] should be studied in task induced adaptation. The pattern of results could potentially shed light on the mechanisms involved. In our opinion, both cases reflect 1) a learning process that reduces a post-saccadic error signal and 2) a learning process that relies on an adaptive adjustment of the motor behavior.

As we did not measure object localization during the current study, we analyzed saccade curvature to gain insights about the mechanisms involved in the oculomotor learning process. An increase in saccade curvature can indicate adaptation of the forward model, but can also indicate the presence of a distractor. In the first case, the saccade first goes toward the initial target location and then, toward the end of the movement, is corrected toward the stepped target location. This correction of the late saccade trajectory results in curvature [56]. In the second case, the saccade trajectory curves away from a distractor. This phenomenon, known as oculomotor inhibition, occurs when a salient distractor competes with the target object. If sufficient resources and time are available to resolve the conflict between the distractor and the saccade target object, the saccade trajectory curves away from the distractor, leading to a deviation from a straight line between saccade start and end point. If the conflict between competing objects is not resolved before saccade onset, then the saccade either lands in between distractor items, or it curves toward the distractor [48,54,55]. In the current study, the curvature index indicates that the saccade trajectory deviated counterclockwise from a straight line once feedback was introduced, suggesting that saccades curved away from the intended target object. Although we cannot exclude that adaptive changes to the forward model developed simultaneously, we conclude that adaptation of the oculomotor behavior to the demands of the task relied primarily on changes to target selection.

The feedback type also had no effect on primary saccade latency. However, saccade latency was influenced by the learning phase. More specifically, saccade latency increased from the pre learning phase, during which no feedback was presented and no manipulation applied, to the early learning phase, where feedback and manipulation were introduced. It is likely that the increase in saccade latency reflects part of the learning process that occurred during the experiment: Once the participants were presented with the distorted feedback, they 1) had to understand the nature of the manipulation (i.e. a constant clockwise 45 arc deg rotation) and 2) to learn a new contingency between action and consequence (i.e. between oculomotor behavior and task success). The corresponding cognitive load might have led to the increase in saccade preparation time.

The angle between the eye position following secondary saccades and the intended target changed throughout the experiment. During the pre-learning circle-off trials, the angle was not different from zero, indicating that those secondary saccades were made to reduce the distance between the eye and the intended target object. Once the distortion and feedback were introduced during the early learning phase, the angle between eye position and intended target object began to increase, corresponding to a decreasing angle between eye position and optimal point. The angle between eye position and intended object continued to increase during the late learning phase, but decreased significantly once the feedback was removed, even though not returning to the baseline level. These results support the finding that participants in our study learned to make their saccades toward the optimal point to achieve task success. The optimal point might have become the goal for the oculomotor system, and the secondary saccades that followed the primary saccades to the optimal point were to further reduce the distance between the fovea and the saccade goal. It is especially remarkable that the secondary saccades were made toward the optimal point given the high salience of the visual feedback signal that was added to the post-saccadic scene. Participants even had to shift their attention to the feedback signal to evaluate their task performance and in doing so the need for adjustment of the oculomotor behavior. Yet, this shift of attention is not reflected in primary or secondary saccade targeting. These results indicate that the feedback signal, even if it attracted attention, did not prevent that saccades were made toward the optimal point and are thus in line with previous research demonstrating that adaptation goes for the goal, even in the presence of distractors [31].

Taken together, our findings suggest that both motor and action feedback were similarly effective. Thus, information about the decoded action and information about the movement that serves as input for the action decoder both foster the understanding of the adaptive adjustment required, and both can be used equally well to adapt the oculomotor behavior to task demands. It suggests that the task (to select the intended object) might have rendered certain features in the visual image relevant (the feedback signal), independent of the exact nature of the feedback signal. This finding is in line with previous research demonstrating that different features or signals in the post-saccadic image can guide post-saccadic error evaluation, depending on the task [26]. Our results thus support the close link between oculomotor control and current tasks [2,3,24,25], as well as between adaptation and achieving task goals [26,28,29,72]. Further, they add to the evidence that eye movements can be used to infer object selection, and by extension, action intentions [2–9]. Further, they indicate that adaptive changes to the gaze behavior can be induced by artificial post-saccadic feedback signals. The results of the current study also add to the research stating that eye movements are a suitable means to communicate action intentions to a human machine interface and thus could be used as input to assistive devices for patients with motor impairment [36,37] as the participants in our study learned to successfully communicate their intended object with their gaze.

Overall, our results suggest that artificial and externally attributed post-saccadic feedback signals can be used to adapt oculomotor behavior to task demands. Both feedback about the executed eye movement and feedback about the decoded intention derived from the eye movement were equally suitable to induce learning, and task performance improved regardless of the exact nature of the feedback signal. Furthermore, participants in our study were able to communicate an intended target object to a simulated system and were even capable of adapting their oculomotor behavior to an externally caused error, i.e. the error was inherent to the system. This finding underlines that gaze signals are a promising candidate for communication with a human-machine interface. Such communication can enable e.g. patients with spinal cord injury to operate an assistive device that compensates for the motor impairment, allowing them to perform everyday tasks independently.
